# Structural and Biochemical Analysis of a Unique Phosphatase from *Bdellovibrio bacteriovorus* Reveals Its Structural and Functional Relationship with the Protein Tyrosine Phosphatase Class of Phytase

**DOI:** 10.1371/journal.pone.0094403

**Published:** 2014-04-09

**Authors:** Robert J. Gruninger, John Thibault, Michael J. Capeness, Robert Till, Steven C. Mosimann, R. Elizabeth Sockett, Brent L. Selinger, Andrew L. Lovering

**Affiliations:** 1 Lethbridge Research Center, Agriculture & Agri-Foods Canada, Lethbridge, AB, Canada; 2 Centre for Genetics and Genomics, School of Life Sciences, University of Nottingham, Medical School, QMC, Nottingham, United Kingdom; 3 Department of Biological Sciences, University of Lethbridge, Lethbridge, AB, Canada; 4 Department of Chemistry and Biochemistry, University of Lethbridge, Lethbridge, AB, Canada; 5 Institute of Microbiology and Infection & School of Biosciences, University of Birmingham, Birmingham, United Kingdom; University of Strathclyde, United Kingdom

## Abstract

*Bdellovibrio bacteriovorus* is an unusual δ-proteobacterium that invades and preys on other Gram-negative bacteria and is of potential interest as a whole cell therapeutic against pathogens of man, animals and crops. PTPs (protein tyrosine phosphatases) are an important class of enzyme involved in desphosphorylating a variety of substrates, often with implications in cell signaling. The *B. bacteriovorus* open reading frame Bd1204 is predicted to encode a PTP of unknown function. Bd1204 is both structurally and mechanistically related to the PTP-like phytase (PTPLP) class of enzymes and possesses a number of unique properties not observed in any other PTPLPs characterized to date. Bd1204 does not display catalytic activity against some common protein tyrosine phosphatase substrates but is highly specific for hydrolysis of phosphomonoester bonds of inositol hexakisphosphate. The structure reveals that Bd1204 has the smallest and least electropositive active site of all characterized PTPLPs to date yet possesses a unique substrate specificity characterized by a strict preference for inositol hexakisphosphate. These two active site features are believed to be the most significant contributors to the specificity of phytate degrading enzymes. We speculate that Bd1204 may be involved in phosphate acquisition outside of prey.

## Introduction

Phytases are a group of enzymes that catalyze the release of orthophosphate from Inositol hexakisphosphate (InsP_6_) [1]. There are four classes of phytases that have been characterized to date, including the histidine acid phosphatases (HAP), β-propeller phytase (BPP), purple acid phosphatases, and the protein tyrosine phosphatase-like phytases (PTPLPs), also known as cysteine phytases [1], [2], [3]. An examination of the phytase diversity within microbial genomes and environmental metagenomes revealed that the BPP class plays a major role in phytate degradation in aquatic environments whereas HAPs were predominant in enteric bacteria and plant pathogens [4]. Proteins belonging to the PTPLP superfamily are prevalent within anaerobic bacteria that inhabit the gut, particularly members of the *Firmicutes* phylum [3], [4], [5]. Proteins belonging to the PTPLP superfamily have also been identified in the plant pathogens *Pseudomonas syringae* and *Xanthomonas campestris,* mammalian pathogens *Clostridium botulinum* and *Legionella pneumophila*, the myxobacterium *Stigmatella aurentiaca* and the predatory bacterium *Bdellovibrio bacteriovorus*. The latter are predatory bacteria that penetrate the periplasmic space of other Gram-negative bacteria, metabolizing the cytoplasmic and periplasmic contents of prey from within before reproducing and eventually lysing the prey cell [6]. The presence of a PTPLP encoding gene in *B. bacteriovorus* raised interesting questions about the possible role of this enzyme in the biology of this predatory species, particularly since InsP_6_ is more likely to be encountered outside of prey in the environment [7].

Thus far, the only characterized PTPLPs have been from anaerobic bacteria [2], [8], [9], [10]. *In vitro*, these enzymes have been shown to hydrolyze InsP_6_ to less-highly phosphorylated IPPs; thereby increasing the availability of free inorganic phosphate [2], [8], [9], [10]. Starting from these contextual observations, we undertook a series of structural, functional and in-vivo experiments aimed at determining whether Bd1204 encodes a “true” phytase.

## Materials and Methods

### Cloning and Mutagenesis

The *B. bacteriovorus* HD100 open reading frame Bd1204 (minus putative signal peptide, amino acids 1–18 as identified by the SignalP server [11]; numbering relates to the complete coding sequence of Bd1204 (**CAE79111**)) was amplified from genomic DNA using polymerase chain reaction (PCR) with Phusion DNA polymerase (Finnzymes) using the forward and reverse primers 5′-**CAT ATG** CAA AAA TCC GTA AGC CTC ACT-3′ and 5′- **CAT ATG** TCA TCT TAA CAC CCA CTC ACC-3′, respectively. An *Nde*I restriction site (bold) was introduced into the primers for cloning purposes. The PCR product was digested with NdeI, ligated into pET28b (Novagen) and transformed into *Escherichia coli* DH5α cells (Novagen) according to manufacturer’s protocol. Mutagenesis was carried out using the Phusion Site-Directed mutagensis kit (Finzymes) according to manufacturers instructions.

### In vivo Studies of Bd1204

The *bd1204* gene was deleted to leave the first two and the last three codons in frame, using reciprocal recombination of a construct conjugated into *B. bacteriovorus* HD100 from suicide vector pK18mobsacB, and exconjugants were screened by Southern blotting and reverse transcription (RT)-PCR to verify the gene deletion. The mobilizable vector pSUP404.2 was used to express Bd1204 with 200 bp of up- and downstream DNA, in *B. bacteriovorus* for complementation studies. RT-PCR with *bd1204-*specific primers was used to confirm gene expression during complementation by this plasmid construct, and RT-PCR by standard methods was used to detect the expression of Bd1204 during predatory and axenic growth in wild type *B. bacteriovorus* HD100 preying upon *E.coli* S17-1. The predicted open reading frame of Bd1204 without the stop codon was fused to the mCherry gene with a five amino acid linker, and this construct was cloned into pK18mobsacB. This was conjugated into B. bacteriovorus HD100 to recombine with the genomic copy of the gene, resulting in a tagged gene with the natural promoter and a promoterless second copy of the gene. Kanamycin selection for pK18mobsacB, which cannot replicate autonomously, maintained this construct in the genome.

### Purification of Bd1204

*Escherichia coli* BL21(DE3) cells were transformed with the pET28b expression construct and grown to an optical density (600 nm) of 0.6–0.8 in Luria-Bertani broth supplemented with 50 μg/mL of kanamycin. Protein expression was induced by adding isopropyl-β-D-thiogalactopyranoside to the culture at a final concentration of 1 mM. The cells were incubated at 37°C with shaking for 18 h before harvesting. Pelleted cells were resuspended in lysis buffer (20 mM KH_2_PO_4_ (pH 7.0), 300 mM NaCl, 10 mM β-mercaptoethanol (βME), 25 mM imidazole (pH 8.0)) and lysed via sonication. Cell debris was removed from the resulting cell lysate by centrifugation. Bd1204 was purified to homogeneity via a combination of affinity and size-exclusion chromatography (using a 5 ml HiTrap Ni-IMAC and superdex 200 column, respectively). Protein concentration was determined by measuring absorbance at 280 nm using the extinction coefficient as calculated by PROTPARAM [12]. The purified protein was stored in 10 mM KH_2_PO_4_ (pH 7.0), 100 mM NaCl, 10 mM βME, and 0.1 mM EDTA. Purified protein was used immediately, or flash frozen in liquid nitrogen and stored at −80°C.

### Crystallization

Crystallization experiments were conducted using sitting drop vapor diffusion with a drop ratio of 1 μl of 20–30 mg/ml protein solution and 1 μl of reservoir. Crystals were grown at 295 K, in 200 mM MgCl_2_, 100 mM Tris-HCl (pH 7.0), 8–10% PEG 8000. Crystals were cryo-protected by gradually transferring them into a solution containing the crystallization reagents and 22% w/v glycerol, followed by flash cooling in liquid nitrogen.

### Data Collection and Structure Determination

Data was collected on beamline 08ID-1 at the Canadian Light Source at a wavelength of 0.97949 Å. Diffraction data was integrated with XDS [13] and scaled with Scala and the CCP4 suite of programs [14]. The structure was solved by molecular replacement with Phaser-MR in Phenix [15] using the phytase from *Selenomonas ruminantium* (PDB 2B4U) as a search model (37% sequence identity). The resulting electron density map was used for automated side chain building with ARP/wARP [16]. This initial model served as a starting point for iterative cycles of TLS, positional, real space and B-factor refinement in Phoenix followed by manual model building with Coot [17]. Statistics for the data collection and refinement are shown in Table 1.

**Table 1:** Data collection and refinement statistics^1^.

	Native
Data collection	
Space group	P2_1_
Unit cell a, b, c (Å)/β(°)	53.6, 78.6, 69.8/β = 93.1
Wavelength (Å)	0.97949
Resolution (Å)	34.2–1.70
Observed reflections	213256 (27255)
Unique reflections	63333 (8858)
Completeness (%)	99.2 (94.7)
Redundancy	3.4 (3.1)
R_merge_	0.078 (0.433)
I/σI	7.2 (2.1)
Refinement Statistics	
Resolution (Å)	34.2–1.70
No. reflections work set	60,097
No. reflections test set	3,211
R_work_ (%)	15.6
R_free_ (%)	19.3
Protein atoms	4313
Solvent atoms	578
Wilson B (Å^2^)	24.0
Average B protein (Å^2^)	30.9
Average B solvent (Å^2^)	38.0
RMSD Bonds (Å)	0.017
RMSD Angles (°)	1.65
Ramachandran distribution	
Most favored (%)	98.4
Additionally allowed (%)	1.6

1 values in parenthesis are for the highest resolution shell (1.79–1.70 Å).

### Analysis of Enzymatic Activity

Enzyme assays were conducted at 50°C (the optimum temperature of the enzyme) unless specified otherwise. Enzyme reaction mixtures consisted of a 600 μL buffered substrate solution (2 mM phytate in 50 mM sodium acetate pH 4.0) and 150 μL of 99.7 nM enzyme solution. The ionic strength (*I*) of the solution was held constant at 50 mM with the addition of NaCl, or when examining the effects of *I*, varied from 0.05 to 0.8 M by increasing the concentration of NaCl. To determine the effect of pH, activity was measured at the following pH ranges with overlapping buffer systems: glycine (pH 3), formate (pH 3–4), acetate (pH 4–6), and Tris-HCl (pH 6–7). To examine the effect of temperature on enzyme activity the temperature of the assay was varied from 25°C to 60°C in 5°C increments. Steady state kinetics were carried out by varying the concentration of sodium phytate between 0.006–0.800 mM in the standard enzyme assay. Steady state kinetic data for the hydrolysis of InsP_6_ were fitted to the Michaelis-Menten equation using non-linear regression (Sigma-plot 8.0; Systat Software Inc.) Sodium phytate was replaced with other phosphoester containing substrates for the determination of substrate specificity. All substrates tested were purchased from Sigma-Aldrich. Enzyme reactions were incubated for 3 minutes at which point the reaction was stopped by the addition of 750 μL of 5% (w/v) trichloroacetic acid, followed by the addition of 750 μL of phosphomolybdate coloring reagent. The coloring reagent was prepared by the addition of 4 volumes 1.5% (w/v) ammonium molybdate solution in 5.5% (v/v) sulfuric acid to 1 volume 2.7% (w/v) ferrous sulfate solution. Liberated inorganic phosphate was measured via A_700_ spectrophotometer readings. To quantify the released phosphate, a calibration curve was produced for each quantification method over a range of 5–600 nmol phosphate/2 mL reaction mixture. Activity (U) was expressed as μmol phosphate liberated per min. Blanks were prepared by addition of the stop solution to the assay mixture prior to addition of the enzyme solution.

### Protein Structure Accession Code

The structure factors and coordinates for the Bd1204 model have been deposited to the protein databank with the accession code 4NX8.

## Results and Discussion

### Bd1204 Transcription and *in-vivo* Testing Function

**Figure 1:**
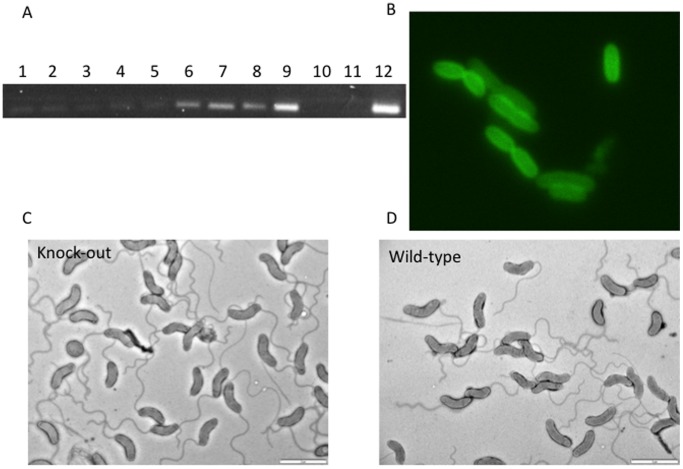
Expression profiling and in vivo testing of Bd1204. A) Transcript profiling of *bd1204* expressed by *B. bacteriovorus* at time points after invasion across the predatory cycle and in host independent (HI) axenic cells. 1– attack phase (no prey), 2–15 min, 3–30 min, 4–45 min, 5–1 hr, 6–2 hr, 7–3 hr, 8–4 hr, 9– HI, 10– no template, 11– *E. coli* S17-1 prey gDNA control–12-HD100 gDNA. B) Expression of Bd1204-mcherry fusion in E. coli shows a periplasmic ring distribution. Comparison of *B. bacteriovorus* cellular structure in C) bd1204 knockout and D) wild type cells.

Transcription of the Bd1204 gene was verified by RT-PCR (Fig. 1a). Transcription of Bd1204 in *B. bacteriovorus* was highest at the late 2–4 h stages of predatory growth and also during axenic (host/prey independent, HI) growth. This result tallies with two transcriptomic studies of *Bdellovibrio*, both of which suggested that Bd1204 transcription is maximal during intra-bdelloplast stages of growth as opposed to prey invasion or attack phase, external prey-seeking times [18], [19]. Fluorescence from a Bd1204-mCherry fusion protein was localized to the periplasm/OM and distributed in a ring around the cell indicating that the putative signal peptide does indeed target the protein for secretion to the periplasm (Fig. 1b). Deletion of the Bd1204 gene did not significantly affect predatory growth or the rate of axenic (HI) growth of the knockout mutant, which was within the range of growth rates of diverse wild-type HI strains. Recently, a protein tyrosine phosphatase homologue in *Caulobacter crescentus* has been found to be important in modulating cell wall synthesis and bacterial cell division [20]. This led us to examine whether Bd1204 may play a similar role in *B. bacteriovorus* by examining the cell structure of the knockout strain by electron microscopy. Comparison of the cell surface of the knockout to wild type cells by electron microscopy did not reveal any unusual features on the cell surface (Fig. 1 c–d) indicating that the protein is not involved in large-scale modifications of the bacterial cell wall. Bioinformatic analysis of a number of predatory bacteria revealed that orthologues of Bd1204 are only found in the closely related *Bdellovibrio bacteriovorus* str. *Tiberius* and the “wolfpack” predatory bacterium *Stigmatella aurantiaca* [21]. Despite being expressed during the later predatory stage of the *Bdellovibrio* life cycle, the lack of conservation of this protein in all predatory bacteria and observed normal predatory function of the knockout strain argue against a role for this protein in predation. To gain insight into the potential function of Bd1204 we carried out a BLASTp analysis and identified the PTPLP from *Selenomonas ruminantium* as its closest characterized homologue (e-value = 4×10^−47^, 37% sequence identity over 243 residues). Importantly, all of the putative catalytic residues are conserved between this *S. ruminantium* phytase and Bd1204, suggesting that Bd1204 may possibly function as a phytase (see kinetic analysis herein).

### Structural Analysis of Bd1204

Bd1204 crystallizes with two copies of the protein in the asymmetric unit. The peptide chain conformations are extremely similar with an RMSD for Cα atoms of 0.31 Å. The buried surface area of the dimer interface was calculated to be 765 Å^2^ using the Protein, interfaces, structures and assemblies web server (http://www.ebi.ac.uk/pdbe/pisa/) suggesting that the observed oligomer is not likely formed in solution. This is confirmed by size exclusion chromatography, which revealed Bd1204 eluting at a volume consistent with a monomer (data not shown). Traceable electron density is observed for amino acids 33–293 in chain A and 33–89, 92–293 in chain B. Residues 19–32 located at the N-terminus are not visible in either chain and presumed to be disordered. A representative figure of the electron density is shown in Figure S1. A DALI search [22] revealed the closest structural homologues to Bd1204 are the PTPLPs from *Selenomonas ruminantium* (PhyAsr; Z-score of 30.2, 37% sequence identity) and *Mitsuokella multacida* (PhyAmm; Z-score 29.9, 35% sequence identity) which is in agreement with the results of a BLASTp search identifying Bd1204 as closely related to the PTPLP superfamily.

**Figure 2:**
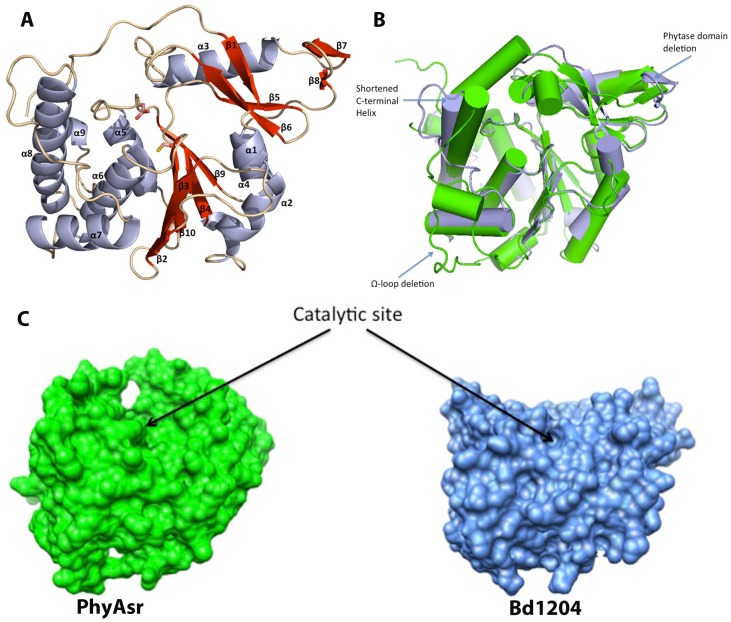
Structure of Bd1204 and comparison to known phytases. A) Bd1204 fold. The phytase domain is composed of strands β1, 5, 6, 7, 8 and helix α3. The catalytic nucleophile (C206) and general acid (D177) are shown as sticks. The phosphate binding loop is located at the center of the active site between β10 and α6. B) Least squares superposition of Bd1204 (light blue) on the *Selenomonas ruminantium* phytase (Green). Significant deletions observed in Bd1204 in the Phytase domain and the Ω-loop are Highlighted. C) Comparison of the surface of the active sites in PhyAsr (Green) and Bd1204. Structures are rotated so that the bottom of the central β-sheet is pointing out of the plane of the page and the phytase domain is into the plane of the page. The surface of Bd1204 is much flatter which results in a much shallower active site relative to PhyAsr.

**Figure 3:**
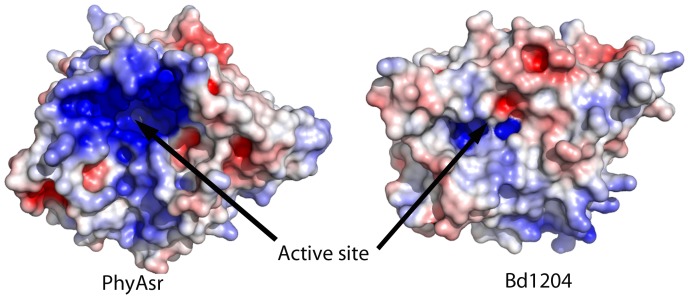
Comparison of the electrostatic surface potential of Bd1204 and PhyAsr. Electrostatic potentials were calculated using the APBS plugin in PyMol. Electropositive regions are colored blue, electronegative regions are colored red and regions of neutral charge are colored white. Structures are oriented to look into the base of the active site with the phytase domain pointing out of the plane of the page.

Bd1204 adopts a canonical PTPLP fold (Figure 2a) with a central 5 stranded mixed β-sheet of topology β2/β3/β10/β4/β9. The central sheet is flanked by 2 α-helices on one side and a 5 helix bundle on the opposite side. An all atom superposition of Bd1204 on the structure of PhyAsr resulted in a root mean square deviation of 1.02 Å indicating that the overall fold of these proteins is very similar (Figure 2b). Although the core fold is conserved between Bd1204 and PhyAsr, there are a number of key differences between these enzymes. The so-called phytase domain [23] that is inserted between β2 and β4 shows some key differences from that present in both PhyAsr and PhyAmm. Importantly, the β-hairpin at the top of the phytase domain between β7 and β8 has a large deletion in Bd1204 that results in the partial β-barrel that is present in both PhyAmm and PhyAsr adopting a much more open conformation (Figure 2). Additionally, a Ω-loop between β2 and β3 that is present in both PhyAsr and PhyAmm is absent in Bd1204. The deletion of this loop results in a significantly shallower active site in Bd1204 relative to PhyAsr, (depth of 7.7 Å versus 14.9 Å, respectively). The linker between α7 and the long C-terminal helix α8 adopts a significantly different conformation in Bd1204 than that observed in PhyAsr, and N-terminal end of the helix is shortened by 2 turns. The combined effect of these structural rearrangements is a much more open, shallower active site relative to both structurally characterized PTPLPs (Figure 2c). This observation is highlighted when the volume and depth of Bd1204 is compared to other members of the PTP family (Table 2). A comparison of the electrostatic surface potential of Bd1204 with PhyAsr reveals a significantly less electropositive active site in Bd1204 (Figure 3). The size and electrostatic potential of the active site has long been suggested to be important in conferring substrate specificity within the PTP superfamily [24]. PTPs that have specificity for phosphoprotein substrates tend to posses shallower active sites whereas enzymes targeting inositol phosphate substrates have deep, positively-charged active sites to enable them to interact effectively with the many highly charged groups on these substrates [2], [23]–[27].

**Table 2:** Variation of active site size and depth in members of the PTP family.

	Volume (Å^3^)	Average depth (Å)
Bd1204	987	7.7
PhyAsr	5370	14.9
PhyAmm D1	4862	13.0
PhyAmm D2	7333	17.0
PTEN	2207	9.8
PTP1B	2049	9.6
VHR	967	8.7

Volume and average depth were calculated using the PDBsum server [34]. PhyAsr – *Selenomonas ruminantium* phytase, PhyAmm D1– N-terminal repeat of *Mitsuokella multacida* phytase, PhyAmm D – N-terminal repeat of *M. multacida* phytase, PTEN - Phosphatase and Tensin Homolog, PTP1B – Protein Tyrosine Phosphatase 1B, VHR - Vaccinia VH1 related dual specificity phosphatase.

**Figure 4:**
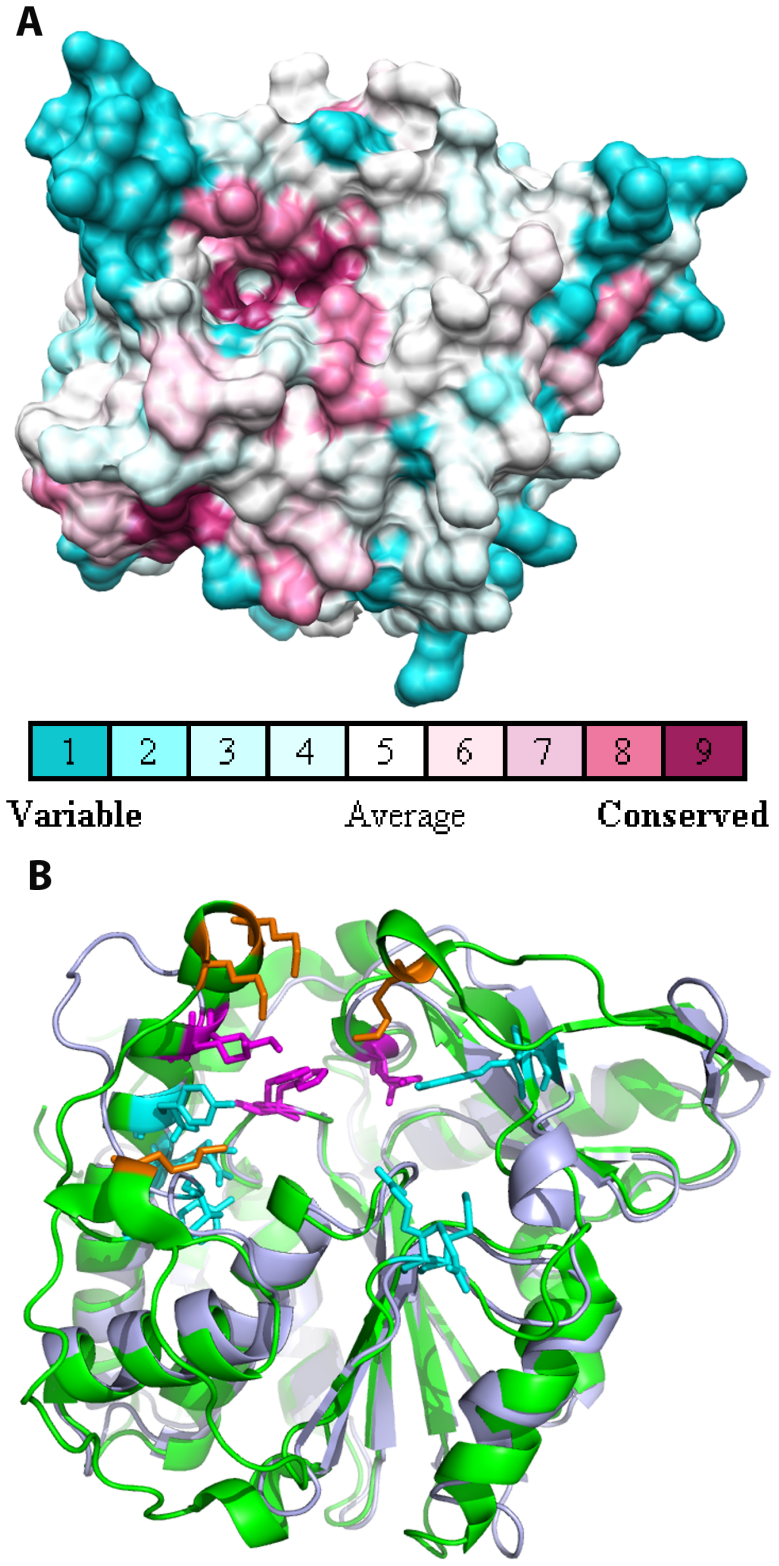
Examination of sequence conservation between Bd1204 and other members of the PTPLP superfamily. A) Consurf analysis of Bd1204 sequence conservation with other members of the PTPLP superfamily. B) Conservation of substrate binding residues in PhyAsr (Green) and Bd1204 (light blue). Residues involved in substrate binding in PhyAsr and the corresponding residues in Bd1204 are shown as sticks. Orange residues have been deleted in Bd1204, cyan residues are not conserved between the two proteins, and magenta residues are conserved. The protein is shown in the same orientation in both panels A and B.

**Figure 5:**
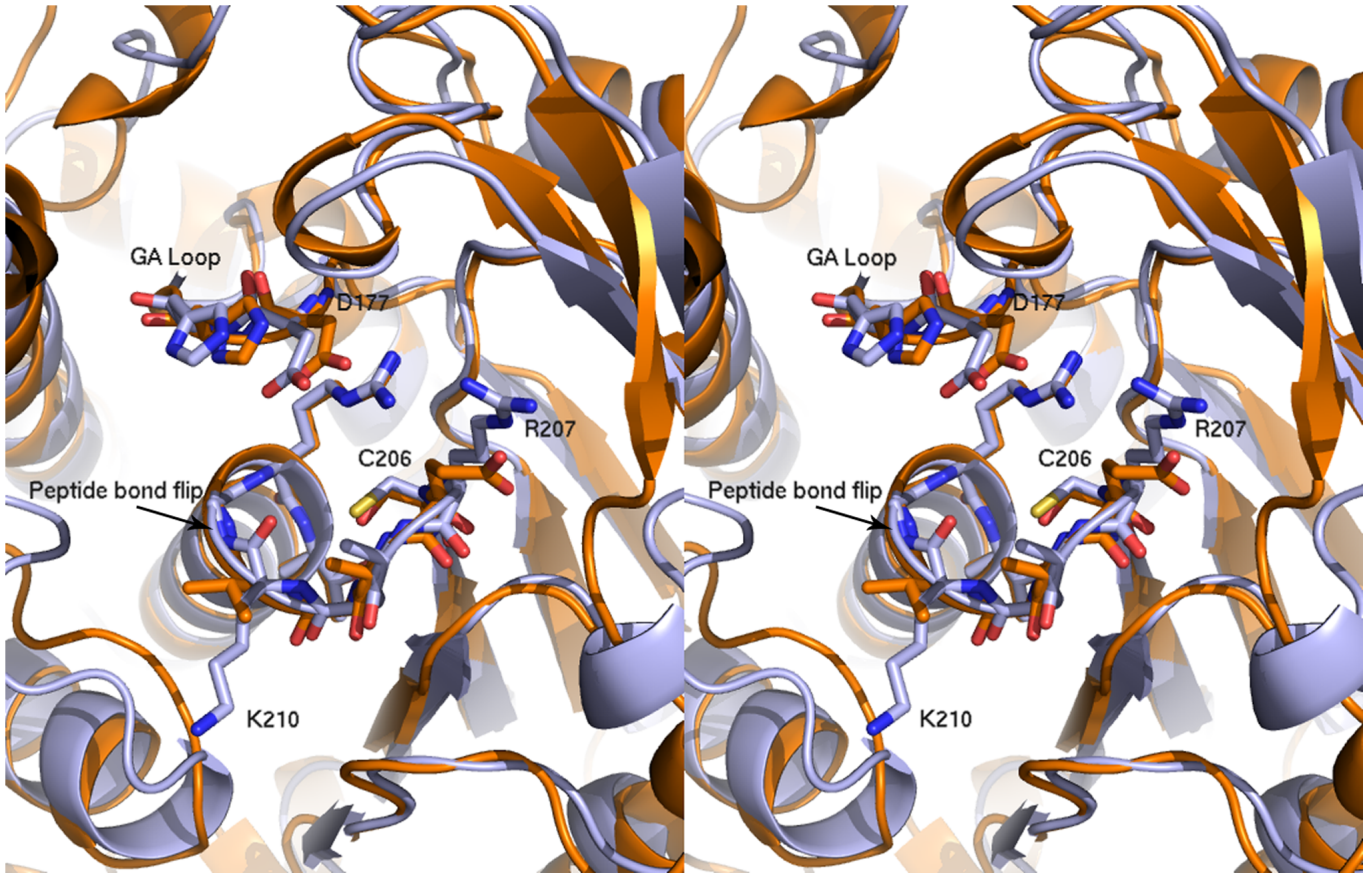
Comparison of the structure of the catalytic site in Bd1204 (Light blue) and PhyAsr (Orange). The position of the catalytic residues, the phosphate binding loop (P-loop) and the general acid loop (GA loop) are conserved in both proteins. Basic residues and peptide bond flip observed in are labeled and shown as sticks. The protein is oriented the same as that shown in Figure 4.

Consurf was used to map sequence conservation onto the structure of Bd1204 [28]. This analysis revealed that the residues that are most highly conserved between Bd1204 and PhyAsr are localized to the immediate vicinity of the catalytic site whilst the regions around the active site, where the enzyme-substrate interactions are localized, are relatively poorly conserved (Figure 4a). A number of residues that are involved in substrate binding in PhyAsr including R57, R68, K83, K189, K299, and K301 are not conserved in Bd1204 (Figure 4b). Many of these residues are localized to the exterior of the binding pocket whereas the residues at the base of the active site show a higher degree of conservation (Figure 4b). These observations are supported by the Consurf analysis and suggest that the specificity of Bd1204 may be altered relative to PhyAsr. Alternatively, there may be compensatory differences such as the presence of R207 and K210 within the phosphate-binding loop (P-loop), which can substitute for the loss of these residues (Figure 5).

Examination of the catalytic motifs reveals that the general acid loop (D_177_H_178_) and the P-loop (HC_206_RAGKGRT) adopt the same conformation in Bd1204 as that observed in PhyAsr and PhyAmm, indicative of a conserved catalytic mechanism (Figure 5). To test this, the putative catalytic nucleophile C206 and putative general acid D177 were mutated to serine and asparagine, respectively. In both cases these mutations abolished catalytic activity (data not shown), indicating that Bd1204 utilizes a classical PTP reaction mechanism. A notable difference within the catalytic site of Bd1204 is a peptide bond flip between K210 and G211 that results in the carbonyl oxygen pointing towards the catalytic cysteine (Figure 5). This peptide bond flip does not alter the P-loop mainchain conformation as was seen upon oxidation of the catalytic cysteine [27]. Examination of the electron density for this residue shows well-resolved density supporting the positioning of the carbonyl oxygen (Figure S1). It is unclear if there is any significance to this or whether there is a conformational change upon substrate binding. Another significant difference between Bd1204 and the PTPLPs characterized to date is the presence of R207 and K210 in the P-loop. R207 is anchored by salt bridges formed with D51 and D120 and occupies the same space in the binding pocket as R57 in PhyAsr, a residue that directly interacts with the substrate. K210 also occupies the space of a residue involved in substrate binding in PhyAsr but that is not conserved in Bd1204. K210 appears to substitute for R68 in PhyAsr. This residue undergoes an induced fit conformational change upon substrate binding. K210 is not anchored and may be able to serve in a similar fashion guiding the substrate into the active site. The inositol phosphatase PTEN also has a basic residue (K128) within its P-loop that is thought to interact with the substrate [29]. A model of InsP_6_ in the active site of Bd1204 based on the complex between PhyAsr and InsP_6_ shows that both R207 and K210 are in a position to interact with phosphates on the substrate. The only contacts <3.5 Å that are made to the modeled InsP_6_ molecule are formed by residues in the general acid loop and the P-loop. The lack of interactions with the modeled substrate suggests that Bd1204 may undergo a structural rearrangement to bind InsP_6_ and/or that InsP_6_ binds in a conformation that is unique from that seen in PhyAsr.

The differences in the size, electrostatic character, and conservation of substrate binding residues between Bd1204 and PhyAsr suggest that the enzymatic properties, particularly the substrate specificity of Bd1204, is altered relative to other characterized PTPLPs. To address this question we have characterized the *in-vitro* enzyme activity of Bd1204.

### Biochemical Characterization of Bd1204 Catalytic Activity

The differences in the structure of Bd1204 in regions of the PTPLP fold that are important in substrate specificity, the altered electrostatic potential, and the decreased depth and size of the Bd1204 active site suggests that this enzyme may have altered substrate specificity relative to other members of the PTPLP superfamily. To examine this hypothesis we carried out enzyme assays with a wide range of phosphate containing substrates to determine if Bd1204 displayed broad or narrow substrate specificity. Surprisingly (given the shallow active site cleft observed in our crystal structure), Bd1204 displays a relatively narrow substrate specificity and is highly active against InsP_6_, showing little catalytic activity against any of the other substrates examined (Table 3). This behavior contrasts with studies of the phytases from *Selenomonas lacticifex* [9], *Selenomonas ruminantium* subsp. *lactolytica* [10], and the N-terminal repeat (D1) of PhyAmm [23] all of which show low sequence conservation in substrate binding regions. Furthermore, at least in the PhyAmm D1 repeat, the structure of these regions and the electrostatic surface potential of the active site are altered relative to the highly specific PTPLPs from *Selenomonas ruminantium* [2], *Megasphaera elsdenii* [8], and the C-terminal repeat of PhyAmm [23]. It had been proposed that PTPLPs that display a broad specificity with low catalytic activity tend to have smaller, less electropositive active sites relative to those that are highly active specific enzymes [23]. It is therefore surprising that Bd1204 displays such a preference for InsP_6_ compared to all other phosphate containing substrates tested.

**Table 3:** Substrate specificity of Bd1204.

Substrate	Specific activity (U mg^−1^)
Ins P_6_	310.78
ATP	1.65
D-fructose 1,6 diphosphate	0.36
α-Naphthyl phosphate	0.20
pNPP	0.14
Phenolphthalein diphosphate	0.093
ADP	0.073
O-phospho-L-tyrosine	0.041
O-nitrophenyl β-D- galactopyranoside	ND
O-phospho-L-threonine	ND
D-fructose 6-phosphate	ND
D-ribose 5-phosphate	ND
D-glucose 6-phosphate	ND
D-glucose 1-phosphate	ND

Substrate concentrations were all tested at 2 mM using the standard enzyme assay described in materials and methods. ND indicates that no catalytic activity was detected.

**Figure 6:**
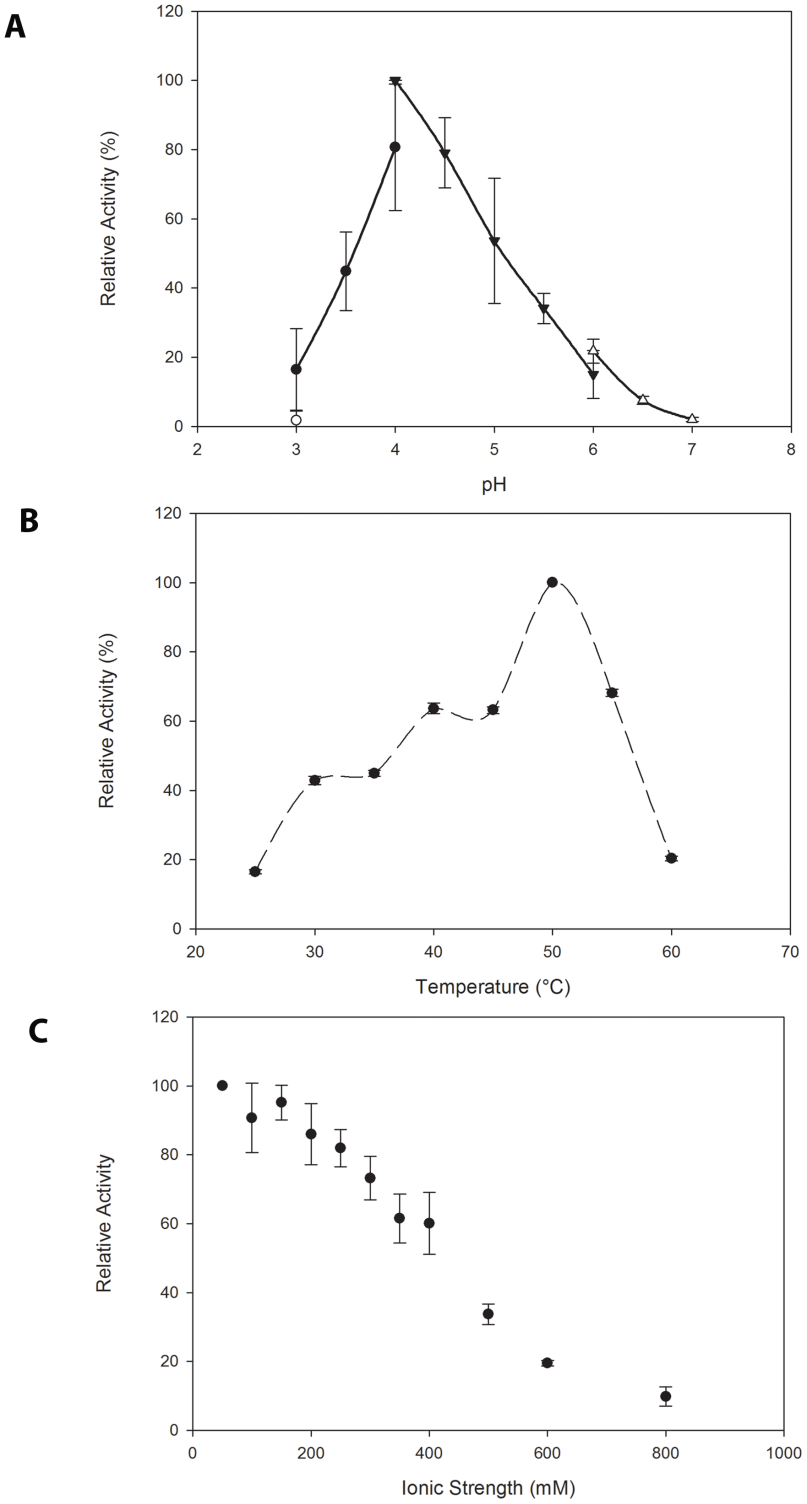
Biochemical characterization of Bd1204. A) pH profile of Bd1204 catalytic activity against InsP_6_. B) Temperature profile of Bd1204 catalytic activity against InsP_6_. C) Effect of increased ionic strength on catalytic activity against InsP_6_.

To gain further insight into the hydrolysis of InsP_6_ by Bd1204, and to classify Bd1204 within the larger group of characterized PTPLPs, we measured the effect of temperature and pH on the rate of InsP_6_ hydrolysis. Bd1204 displays a pH optimum of 4.0 categorizing it as an acid phosphatase (Figure 6a) and a temperature optimum of 50°C (Figure 6b). Based on their pH optima for catalysis, phytases can be divided into two major groups, acid and alkaline [30]. Acid phytases include enzymes belonging to the HAP, PAP, and PTP superfamily classes of phosphatases [1], [2], [8], [9], [10]. Characterization of Bd1204 as an acid phytase adds functional similarity to the structural similarity observed with the characterized PTPLPs from *S. ruminantium*; PhyAsr [2], *M. elsdenii* [8], *S. lacticifex* [9], and *S. ruminantium subsp. lactilytica* [10]. The Bd1204 temperature optima is also similar to that found in the other previously characterized enzymes. Steady state kinetic analysis of Bd1204 displayed typical Michaelis-Menten kinetics with a k_cat_ of 124 s^−1^ and K_m_ of 574 μM for InsP_6_ consistent with other well-characterized PTPLPs. The similarity of the pH, temperature optima, and kinetic constants of these diverse enzymes is somewhat surprising given the vastly different environments they are found in.

An interesting property of PTPLPs is the effect that alterations in ionic strength have on the capability of these enzymes to hydrolyze InsP_6_. It has been shown that this influence is due to the disruption of key ionic interactions between the protein and substrate [2]. The surface charge distribution of the Bd1204 active site is unique in that it is the least electropositive of all currently structurally characterized PTPLPs. To examine the influence that increases in ionic strength have on the rate of InsP_6_ hydrolysis we varied the ionic strength of the reaction buffer and monitored enzyme activity. As with other PTPs and PTPLP enzymes, ionic strength had an effect on the *in-vitro* capabilities of Bd1204 to hydrolyze InsP_6_, revealing an optimal ionic strength of 50 mM, relatively low compared to other PTPLPs characterized to date. Further increases in ionic strength result in a rapid decrease in the relative rate of InsP_6_ hydrolysis that follows an inverse sigmoidal curve (Figure 6c). This rapid decrease contrasts with observations of other PTPLPs, which tend to show significant decreases in catalysis only at very high ionic strengths. It is likely that the dramatic difference in the electrostatic character of the Bd1204 active site accounts for this phenomenon.

## Summary

We have established that the open reading frame Bd1204 in *B. bacteriovorus* encodes a highly specific phytase belonging to the Protein Tyrosine Phosphatase superfamily and that its expression is up-regulated during the late (growth) stages of predation and that it is located in the *B. bacteriovorus* periplasm. We were not able to conclusively identify the *in-vivo* function of Bd1204 but it is possible that it functions in obtaining phosphate from the environment, which may be important in an organism whose usual access to this nutrient is via metabolism of prey. There are several parallel sources of phosphate in prey and uptake of these may preclude a strong phenotype in the *bd1204* deletion strain. A number of phytases in a wide range of environments have been shown to play a role in phosphate uptake [31], [32], [33]. This function is also consistent with the observed secretion of the protein. The role of Bd1204 as a phytase is supported by our biochemical and structural studies of this enzyme. Interestingly, Bd1204 has a number of unique structural features including the smallest and least electropositive active site of any structurally characterized PTPLP to date. Despite this, Bd1204 displays a high degree of substrate specificity and high rate of catalytic activity. This is in contrast not only to all other characterized PTPLPs, but also all other members of the HAP phytase family (this behavior is in direct contrast to what was observed for the HAP from *Aspergillus niger* [25]). These unique biochemical properties of Bd1204 call for a reevaluation of the basis of substrate specificity within this ever-expanding group of proteins.

## Supporting Information

Figure S1 Representative electron density used to model structure of Bd1204. Sigma-A weighted map is contoured at 1.5σ shows the electron density of the active site.
(TIF)
