# Supplementary figures and images for: Structural and Biochemical Analysis of a Unique Phosphatase from Bdellovibrio bacteriovorus Reveals Its Structural and Functional Relationship with the Protein Tyrosine Phosphatase Class of Phytase
Source: PLoS One. 2014 Apr 9;9(4):e94403. doi: 10.1371/journal.pone.0094403 (PMC3981807; doi:10.1371/journal.pone.0094403)

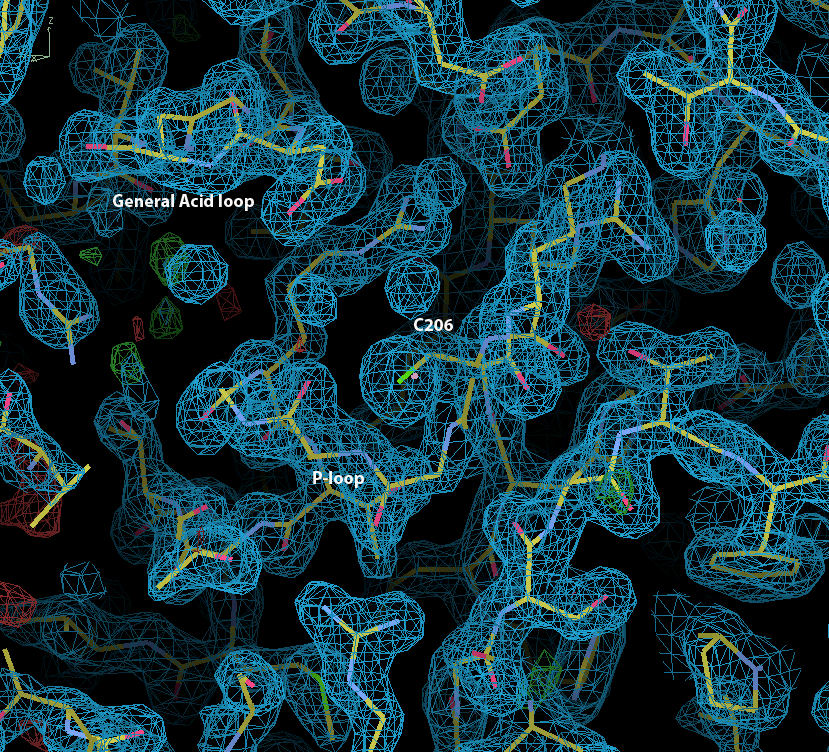

Supplement: Figure S1 — Representative electron density used to model structure of Bd1204. Sigma-A weighted map is contoured at 1.5σ shows the electron density of the active site. (TIF) [file pone.0094403.s001.tif]
